# Etching on the edge: enamel loss under repeated and active HCl applications as a resin infiltration pretreatment

**DOI:** 10.1590/1678-7757-2025-0103

**Published:** 2025-08-29

**Authors:** Maria Paula Novaes Camargo MANNA, Talita Portela PEREIRA, Bruna de Oliveira IATAROLA, Mariele VERTUAN, Ana Carolina MAGALHÃES, Denise Maria ZEZELL, Luciana Fávaro FRANCISCONI-DOS-RIOS

**Affiliations:** 1 Universidade de São Paulo Faculdade de Odontologia Departamento de Dentística São Paulo Brasil Universidade de São Paulo, Faculdade de Odontologia, Departamento de Dentística, São Paulo, Brasil.; 2 Instituto de Ciência e Tecnologia Faculdade de Odontologia Departamento de Dentística São José dos Campos SP Brasil Instituto de Ciência e Tecnologia, Faculdade de Odontologia, Departamento de Dentística, São José dos Campos (SP), Brasil.; 3 Universidade de São Paulo Faculdade de Odontologia de Bauru Departamento de Ciências Biológicas São Paulo Brasil Universidade de São Paulo, Faculdade de Odontologia de Bauru, Departamento de Ciências Biológicas, São Paulo, Brasil.; 4 Universidade de São Paulo Instituto de Pesquisas Energéticas e Nucleares Centro de Lasers e Aplicações São Paulo Brasil Universidade de São Paulo, Instituto de Pesquisas Energéticas e Nucleares, Centro de Lasers e Aplicações, São Paulo, Brasil.

**Keywords:** Dental caries, Acid etching, Hydrochloric acid, Dental enamel

## Abstract

Considering that a single passive application of hydrochloric acid (HCl) as a resin infiltration pretreatment can remove between 20 and 45 µm of enamel and cause etching that extends up to 2 mm beyond the white spot lesions (WSLs), it is plausible that its repeated and active applications could result in a greater amount of dental tissue being removed.

**Objective:**

To evaluate the enamel surface loss and micromorphology after etching with 15% HCl using two application methods (passive-P and active-A) and varying numbers of applications (C-placebo – 120 s; 1x HCl – 120 s; 2x HCl – 120 s + 120 s; 3x HCl – 120 s + 120 s + 120 s).

**Methodology:**

Bovine incisors with ≤0.3 µm initial curvature were randomized into eight groups (n=12) based on microhardness, followed by WSL simulation. A central window was etched according to experimental conditions, and surface loss was assessed using optical profilometry and micromorphology via scanning electron microscopy (SEM). Two-way ANOVA and Tukey's test were used for surface loss, and the chi-square test evaluated the association of experimental conditions with etching patterns (α=0.05).

**Results:**

1xP generated intermediate mean surface loss, positioned between the values observed for passive control (PC) and active control (AC), and those for 2xP and 3xP. Losses from active applications were significantly higher than passive ones and were increased by the number of applications. SEM showed Types II and III etching patterns and Type II was more frequent. There was no association between treatment and etching pattern.

**Conclusion:**

Multiple and active HCl applications may raise concerns about removal of the remaining tooth structure, challenging the principles of minimal intervention dentistry.

## Graphical abstract

**Figure:**
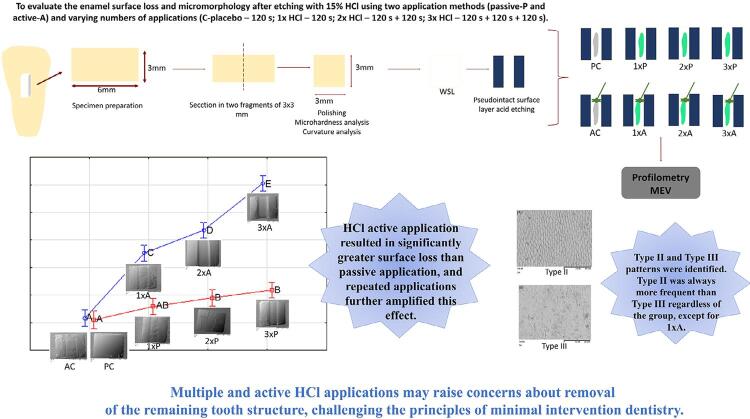

## Introduction

Different approaches are available for the proper management of dental caries and their associated signs.^1^ The management of active and non-cavitated incipient lesions should ideally consist of non-invasive or microinvasive procedures,^1,2^ such as resin infiltration, which not only halts caries progression but also helps to mask the white spot.^3^

The infiltrant, with low viscosity, a high penetration coefficient, and a refractive index relatively close to that of enamel (1.52 vs. 1.62)^4,5^, penetrates into the lesion pores by capillary action,^6,7^ blocking the diffusion pathways of acids produced by bacterial metabolism and minimizing its unaesthetic white appearance.^6,8-15^

Penetration pathways should, however, be created through the pseudointact surface layer, defined as the apparently intact superficial enamel layer with a thickness of 40 μm or less, which overlays the more porous subsurface.^8,9,16^ This should be done according to the recommendations of the manufacturer by applying 15% hydrochloric acid (HCl) (Icon-Etch^®^, DMG, Chemisch-Pharmazeutische Fabrik GmbH, Hamburg, Germany) for 2 min, followed by rinsing and drying.^5,8,17-20^

Around 29% of white spot lesions (WSLs) may have a superficial layer with a thickness greater than 50 μm, making it difficult to create sufficient porosities with just one application of 15% HCl^8^. HCl flow is also impeded by the high surface tension of the gel,^16^ not to mention that air bubbles can prevent its diffusion into some regions of the lesion.^16,20^ Therefore, in some cases, literature^4,17,21^ and the manufacturer considered and recommended repeating the etching procedure once or twice until the white and opaque appearance after drying is at least reduced. Regarding this pre-treatment, other four different factors have been investigated: the type of acid, the duration of acid etching, the method of acid application (with or without brushing), and the use/addition of abrasives.^22^

In this way, the effects of active or passive application of HCl or other acids have been increasingly investigated, both on the penetration of the infiltrant and on the attenuation of the spots.^16,19,20^ However, if pre-treatment with a single passive application can remove up to 70% of the superficial layer^8,16^ or 20 to 45 μm in depth,^8^ and etching can extend up to 2 mm beyond the lesion (Product Insert - DMG), repeated active applications could cause excessive removal of dental tissue.^1^

It has been suggested that resin infiltration is successful only when the entire pseudointact superficial layer is removed, thereby enabling capillary penetration of the material into the deeper regions of the lesion.^6,7,18,16^ Nevertheless, the incomplete corrosion of the pseudointact superficial layer may not hinder infiltrant penetration, and the dark zone with higher organic content is believed to create that problem.^19^ Additionally, when a WSL is still visible on a hydrated tooth (or etched with Icon-Etch^®^ and moistened with Icon-Dry^®^), it has possibly reached the dentin and that may also prevent complete infiltration.^23^

Given the increasing weight placed on minimal intervention dentistry (MID), this *in vitro* study aims to evaluate surface loss and the etching patterns considering passive and active application modes and the number of applications of 15% HCl (Icon-Etch^®^) for the treatment of bovine enamel with incipient carious lesions. The null hypotheses were as follows: 1) the mode of HCl application does not lead to distinct surface loss; 2) the number of HCl applications does not lead to distinct surface loss; and 3) there is no association between enamel treatment and the different micromorphological etching patterns.

## Methodology

### Experimental design

We evaluated the surface loss and micromorphology of bovine enamel with incipient carious lesions etched with 15% HCl. The experimental factors considered were the mode of acid application at two levels (P: passive; A: active) and the number of applications at four levels (C: placebo – 120 s; 1x: 1x HCl – 120 s; 2x: 2x HCl – 120 s + 120 s; 3x: 3x HCl – 120 s + 120 s + 120 s).

Based on previous unpublished data, which showed that losses amounted to 32.37±8.00 µm with HCl etching and to 14.14±1.21 µm with phosphoric acid (H_3_PO_4_) etching, the sample size was calculated using an estimated standard deviation of 8.0, an effect size of 14.0, and alpha and beta errors of 5% and 20%, respectively. The value of n found per group was 11, but we opted for n=12 for safety reasons.

### Specimen preparation

**Figure 1:**
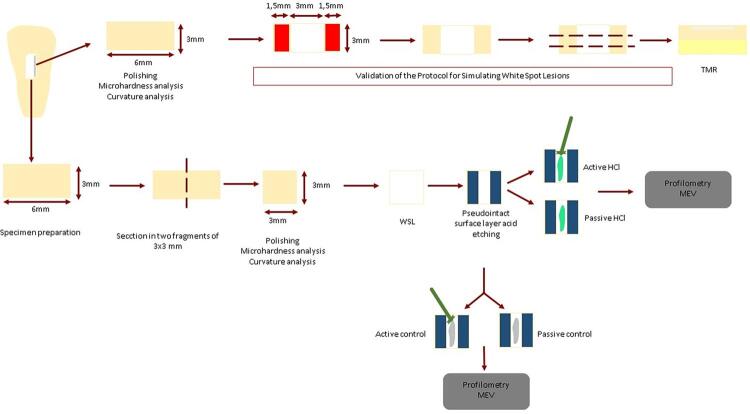
Work Flowchart.

A total of 80 bovine incisors were obtained after exemption of this research project from review granted by the Ethics Committee on Animal Use (CEUA/FOUSP #004/2023). The work flowchart (Figure 1) summarizes the conducted procedures.

The crowns were then sectioned with perpendicular cuts to obtain a fragment measuring 6x3 mm from the flatter central region and two fragments of 3x3 mm after further sectioning,^24^ using a precision cutting machine (Isomet Low Speed Saw; Buehler Ltd., Lake Buff, IL, USA). The dentin and enamel of the fragments were abraded and polished in a metallographic polisher (EcoMet; Buehler Ltd., Lake Buff, IL, USA), and the specimens were then immersed in distilled water for 10 minutes in an ultrasonic bath (Shenzhen Codyson Electrical Co., Ltd., CHN, Guangdong, China).^25^

The specimens were numbered and subjected to an initial curvature analysis using an optical profilometer (Proscan 2100, Scantron, Venture Way, Taunton, UK), and only those with a curvature equal to 0.3 μm or less were used in the study.^26^

All specimens were also subjected to an initial analysis of their surface microhardness (Knoop hardness number [KHN]) using a microhardness tester (HMV-G21DT, Shimadzu Co. Tokyo, Japan) with a Knoop indenter (50 g/10 s).^27^ Five indentations were made to determine the mean and standard deviation of the microhardness value. Blocks with a standard deviation greater than 10% of their individual mean microhardness and individual mean microhardness greater or less than 10% of the mean microhardness calculated for all blocks were excluded.

A total of 96 fragments were selected based on the initial microhardness for distribution, by stratified randomization (Excel 16.0; Microsoft Corporation, Redmond, WA, USA), into the different experimental groups (n=12) (Table 1).

**Table 1:** Experimental groups.

Group	Number of times HCl 15% is applied - 120s	How to apply HCl 15% -120s
AC	Not once (Control – Placebo gel)	Active (A)
PC		Passive (P)
1xA	Once (1x)	Active (A)
1xP		Passive (P)
2xA	Twice (2x)	Active (A)
2xP		Passive (P)
3xA	Three times (3x)	Active (A)
3xP		Passive (P)

### Validation of the protocol for simulating white spot lesions

Nine specimens, measuring 6x3 mm and adequate in terms of initial curvature and microhardness, were selected for pilot studies and prepared with a central window of 3x3 mm. In that window, WSL was simulated by individual immersion and storage of three specimens for 32, 64, or 96 h (n=3), without agitation, at 37°C, in 32 mL of a demineralizing solution consisting of 50 mM acetate buffer containing 1.28 mmol/L of Ca(NO_3_)_2_.4H_2_O, 0.74 mM of NaH_2_PO_4_.2H_2_O, and 0.03 ppm F, at pH 5.0.^11,24,28-30^

The specimens were sectioned into three slices and each measured approximately 0.8 mm in thickness. Each slice was polished manually to obtain 80 to 100 µm slices. They were mounted onto specific plates and subjected to x-ray imaging using transverse microradiography (TMR; TMR 1.25e, Inspector Research BV, Amsterdam, the Netherlands) system. The entire process, including the development and analysis of the images, was conducted as described by Braga, et al.^31^(2018), Souza, et al.^32^ (2018), and Santos, et al.^33^ (2019).

A transmitted light microscope system with a 20x objective (Axioplan; Zeiss, Oberkochen, Germany) and a camera (XC-77CE, Sony, Tokyo, Japan) were used to observe whether there was a subsurface lesion, utilizing the TMR 1.25 system software. The integrated mineral loss (ΔZ, vol.%×μm) was calculated by determining the difference between the mineral volume percentage of sound enamel (87%) and that of demineralized enamel, multiplied by the lesion depth (µm). Additionally, the lesion depth (LD, μm) was defined as the distance from the enamel surface to the point in which the mineral content returned to 95% or more of the healthy enamel value, equivalent to 82.7%. The average mineral loss (R) was then calculated by dividing ΔZ by LD (vol.%) (Table 2).

**Table 2:** Integrated mineral loss (ΔZ, vol.%×μm), lesion depth (LD, µm) and average mineral loss (R,vol.%) of specimens immersed in the demineralizing solution.

Specimen		ΔZ (vol.%×μm)	LD (μm)	R (vol%)
		Mineral Loss	Lesion Depth	Ratio
32 h	6	1645.00	54.93	30.20
	7	2125.00	87.62	24.87
	14	1231.67	43.83	27.73
	Mean	1667.22	62.13	27.60
	SD	447.08	22.76	2.67
64 h	4	2426.00	63.98	38.10
	5	1937.50	70.75	28.88
	12	2794.00	79.96	35.00
	Mean	2385.83	71.56	33.99
	SD	429.66	8.02	4.69
96 h	1	3350.00	87.30	38.30
	18	2008.57	78.47	25.77
	9	2410.00	80.51	29.89
	Mean	2589.52	82.10	31.32
	SD	688.50	4.62	6.39

**Figure 2:**
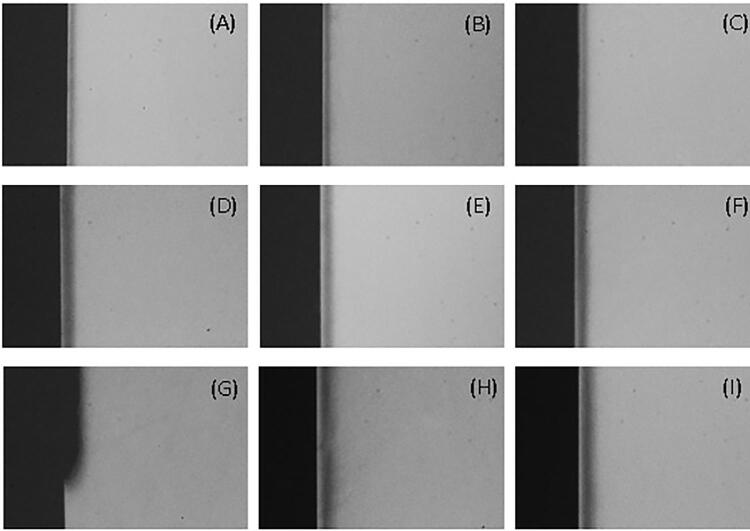
Representative TMR images of most of the slices of specimens demineralized for the times of 32h (A, B, C); 64h (D, E, F) and 96h (G, H, I), with an example of cavitation observed only in image G. *Different letters indicate statistically significant difference between experimental groups

For all immersion times, most of the evaluated specimen slices showed a pseudointact surface layer and typical characteristics of carious lesions (Figure 2), which confirmed that the solution could artificially induce such lesions. No significantly greater lesion depth was observed after 96 h compared to 64 h, and the subsurface lesion pattern with preservation of the surface layer was more specifically/frequently observed at 64 h.

Therefore, a 64-hour immersion time was chosen for simulating WSLs in the specimens of the main study, using the same solution prepared for validation.

### Simulation of white spot lesions

The lesions were then created over the entire enamel surface, following the validated protocol, using 64 h as immersion time.

### Pseudointact surface layer acid etching

The central window (1x3 mm) was then rinsed with an air-water spray for 30 s and dried with an oil/water-free air spray from a triple syringe for another 30 s. The control group specimens were etched with a placebo gel prepared in the lab (100 mL of distilled water and 20 g of hydroxypropyl methylcellulose)^34^ for 120 s. Either were actively applied, by maintaining constant friction with a disposable microbrush (Cavibrush Extra Fine Disposable Micro Applicator; FGM Produtos Odontológicos, Joinville, SC, Brazil) throughout the application, or passively, enabling it to rest for the designated time. Subsequently, the area was thoroughly rinsed for 30 s with an air-water spray, followed by vigorous drying with an air spray for 30 s, always maintaining approximately 1 cm from the surface.

The treatment group specimens had the window etched with 15% HCl (Icon-Etch^®^ - DMG, Chemisch-Pharmazeutische Fabrik GmbH, Hamburg, Germany – donated by the manufacturer, batches 794250, 794324, 250853, and 250857) for 120 s, which were either applied actively or passively once, twice, or three times, as described for the predefined groups. Sixteen specimens — two from each group — were processed daily in a rotating order to minimize fatigue effects. All procedures were conducted by a single calibrated operator, which received training to apply consistent pressure and maintain proper brush positioning during active acid etching.

### Surface loss analysis by profilometry

A central area of 2 mm in length (x-axis) by 1 mm in width (y-axis) of each specimen was scanned by the optical profilometer to cover the treated and reference areas on either side. The equipment was set to travel 200 steps of 0.01 mm on the x-axis and 10 steps of 0.1 mm on the y-axis. The surface loss (in μm) of etching was calculated by the software based on the subtraction of the mean height of the test area from that of the two reference areas.

### Surface micromorphology analysis by scanning electron microscopy

The surface micromorphology of the etched enamel was analyzed using micrographs of the most representative regions of the specimens, under a bench scanning electron microscope (Hitachi Analytical Table Top Microscope TM3000, Hitachi, Tokyo, Japan), operating at 5 Kv, with 2000x and 4000x magnification. Each specimen was classified according to the typical etching patterns of the enamel (Type I, Type II, or Type III) to determine its frequency for each experimental group based on the surface micromorphology. When there were different etching patterns in the same specimen, the classification of the most prevalent pattern was assigned.

### Statistical analysis

The two-way analysis of variance (ANOVA) was applied to detect the effects of the etching mode (passive/active) and the number of applications (C, 1x, 2x, or 3x) on surface loss, followed by Tukey’s *post-hoc* test to identify differences between the groups. The micromorphology data were analyzed using a chi-square test to detect differences between the etching patterns that the experimental groups showed. Significance level was always 0.05, and Statistica (13.5.0.17, TIBCO Software Inc., Palo Alto/CA, USA) was used for the statistical analyses.

## Results

### Surface loss

**Figure 3:**
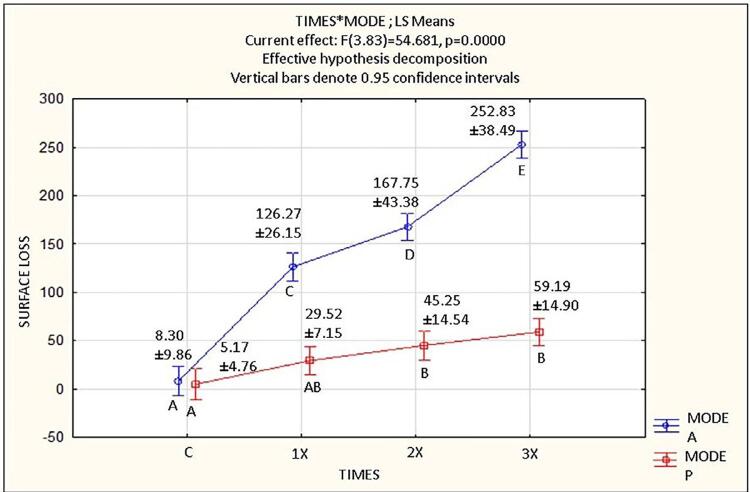
Mean±standard deviation values of surface loss (depth - µm) as a function of the application mode (A: active; P: passive), and number of applications of 15% HCl gel (C: placebo gel; 1x: once; 2x: twice; 3x: three times).

Both experimental conditions, the number of applications (C, 1x, 2x, or 3x) (p<0.001), and the application method (A or P) (p<0.001) had a significant influence on surface loss results, and there was a significant interaction between these factors (p<0.001). Figure 3 shows the mean values and standard deviations of surface loss as a function of the application method and number of applications.

There was no statistically significant difference between PC, AC, and 1xP, nor between 1xP and 2xP and 3xP. Thus, note that 1xP presents intermediate values of surface loss, positioned between values observed for PC and AC, and those for 2xP and 3xP.

Moreover, all values for surface losses from active applications (1xA, 2xA, and 3xA) were statistically higher than any of the losses from passive applications and the active control. Additionally, the loss increased gradually with the number of applications (3xA>2xA>1xA), and the highest surface loss was observed for 3xA, which was 1.5 times greater than the loss of 2xA and two times greater than the loss of 1xA. Surface loss in 3xA was 4.27 times greater than 3xP; in 2xA, it was 3.7 times greater than 2xP; and in 1xA, it was 4.27 times greater than 1xP. All of these values were statistically significant.

### Surface mictomorphology

**Figure 4:**
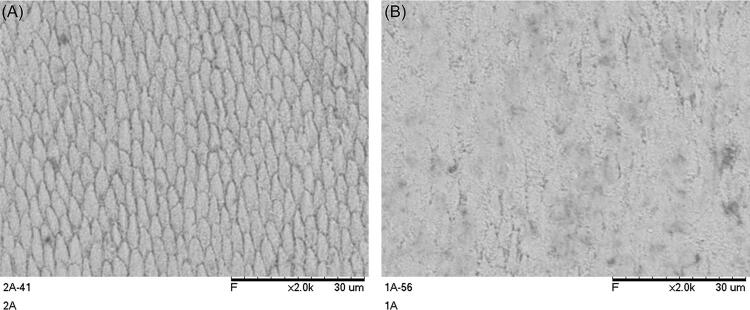
Different etching patterns observed; A - Type II (prism peripheries dissolution); B - Type III (a I and II mixed or irregular pattern not clearly following prism boundaries).

Regarding the three classic etching patterns (Type I - prism core dissolution, Type II - prism peripheries dissolution, Type III - a I and II mixed or irregular pattern not clearly following prism boundaries)^35,36^only Type II and Type III were identified (Figure 4).

The chi-square test for the etching patterns revealed no association between etching pattern and acid application method (p=0.536). However, numerically and overall, Type II pattern was always more frequent than Type III regardless of the group, except for 1xA (Table 3).

**Table 3:** Frequency of etching patterns observed by group.

TREATMENT	Frequency	Type II	Type III
1xA	n	6	6
	%	50.00%	50.00%
1xP	n	7	5
	%	58.33%	41.67%
2xA	n	10	2
	%	83.33%	16.67%
2xP	n	7	5
	%	58.33%	41.67%
3xA	n	9	3
	%	75.00%	25.00%
3xP	n	7	5
	%	58.33%	41.67%

**Figure 5:**
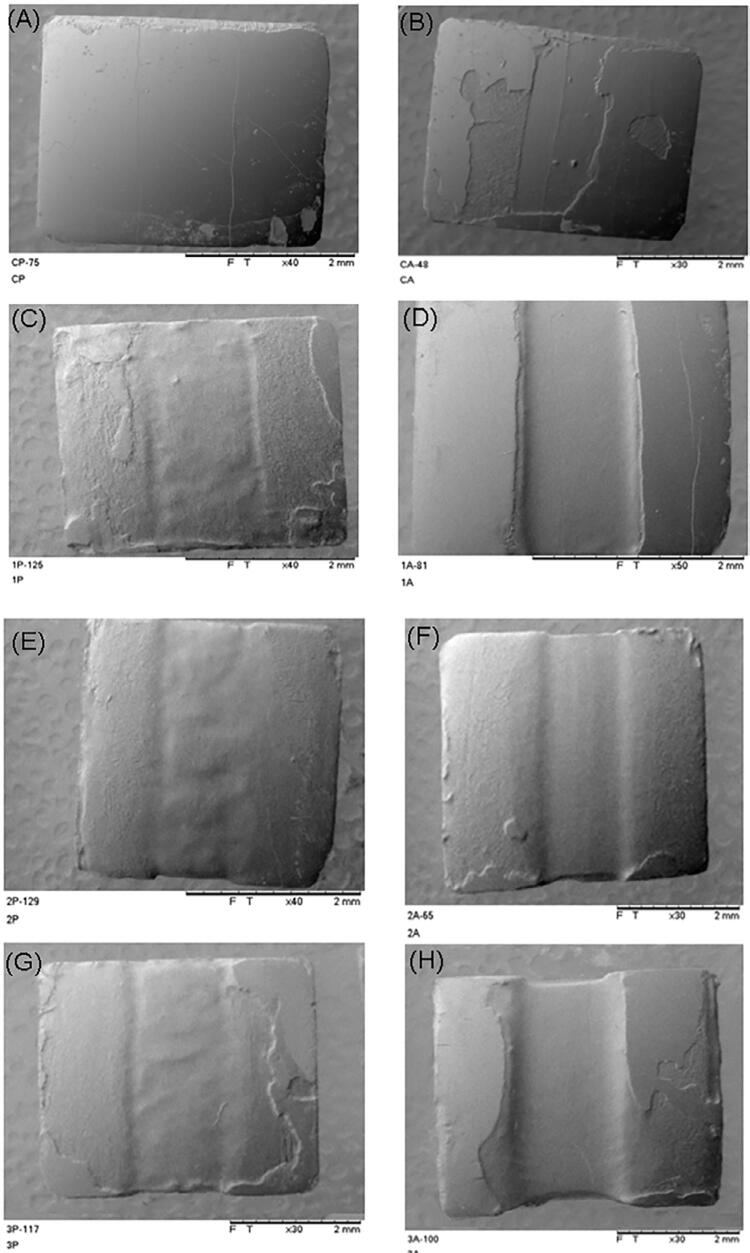
Visualization of the surface topographies observed under scanning electron microscopy, according to the different surface treatments employed (A - passive control; B - active control; C - 1x passive; D - 1x active; E - 2x passive; F - 2x active; G - 3x passive; H - 3x active).

**Figure 6:**
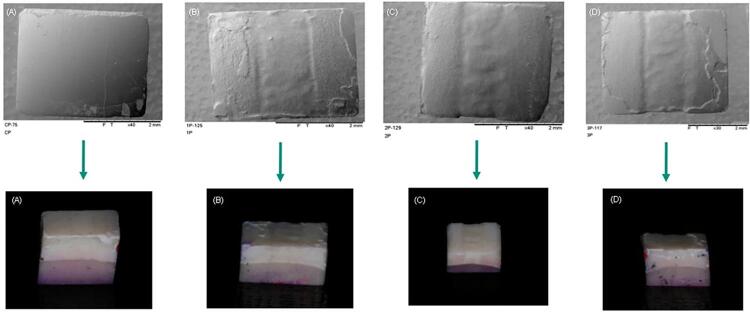
Comparison of images obtained by SEM and digital camera (A - passive control; B - 1x passive; C - 2x passive; D - 3x passive).

**Figure 7:**
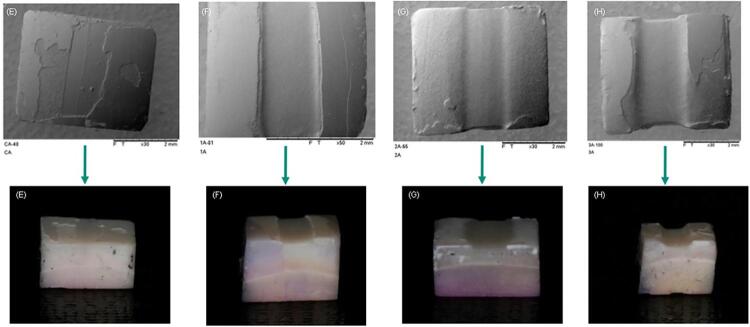
Comparison of images obtained by SEM and digital camera (E - active control; F - 1x active; G - 2x active; H - 3x active).

Figures 5, 6, and 7 show that it became possible to verify the different consequences of the various treatments via topographic images of the specimens and the digital camera. There was no loss observed in most cases of the images related to the PC group, however, it became possible to visualize some surface loss, which is possibly due to technical artifacts, produced by either specimen handling or even washing pressure. Visible loss was observed in the CA group due to the friction imposed by the microbrush during the 120 s, and the losses gradually increased with the number of applications in the treatment groups, and they were even greater in the active treatment groups.

## Discussion

Based on those findings, the first two null hypotheses were rejected, as the method of HCl application and the number of applications both significantly influenced surface loss.

In agreement with the results, previous research reported an average surface loss of 34.02 μm following a 2-minute passive application of 15% HCl on sound human enamel, while four applications increased the depth to 77.56 μm.^21^ Thus, while a single passive application following the original manufacturer’s instructions typically results in approximately 30 μm of surface loss — generally within the mean thickness of the non-cavitated surface layer — multiple applications may lead to removals that could exceed the pseudo intact superficial layer,^21^ causing cavitation and thus potentially undermining the intended indication of the Icon^®^ system.

Note that these results were obtained on sound enamel and under passive application conditions, in contrast to this study, which evaluated white spot lesions and included active acid application. Actively applying HCl three times resulted in a surface loss of 252.83 ± 38.49 μm, such as that produced by microabrasion, even in the absence of an abrasive agent. Tong, et al.^4^ (1993) reported that direct application of 18% HCl on sound enamel caused a 100 ± 47 μm loss, whereas combining it with pumice resulted in 360 ± 130 μm loss. Another study likewise reported that 15% HCl applied passively for 120 seconds resulted in enamel loss of only 30-40 μm, whereas microabrasion removed approximately 360 μm after twenty applications of five seconds.^37^ The results of the present work thus underscore the critical role of friction — even in the absence of an abrasive agent — in accelerating enamel removal. Multiple active HCl applications can severely compromise enamel structure and potentially remove entire lesions, depending on their depth.

Considering this, previous studies have explored whether alternative etching approaches — such as the mechanical application of milder acids — might enhance surface permeability and promote resin infiltration, even with reduced surface loss. One of them showed that passive 15% HCl application for 120 seconds on natural WSLs produced approximately 2.7 times more surface loss than 30 seconds of H₃PO₄ applied with friction (36 ± 7.62 μm vs. 13 ± 2.76 μm; p < 0.001) .^38^ Although the depth of surface removal was significantly lower with mechanical friction using H₃PO₄, the surface permeability increased due to micromorphological changes, which led to comparable degrees of resin penetration between groups.^38^ Such findings suggest that surface permeability enhancement, rather than depth of surface removal alone, may be critical for successful infiltrant penetration, in which factors such as enamel morphology and infiltration time also play influential roles.^38^

However, the manner in which acid is applied with friction appears to warrant further attention, as Yim, et al.^39^ (2014) highlights in observations of artificial lesions prior to their investigations on natural ones.^38^ Although this was not the focus of this study, their findings showed that friction-based application of 37% H_3_PO_4_ with a brush resulted in surface loss comparable to passive 15% HCl, while also increasing pore volume and requiring shorter application time (120 *vs*. 30 s). In contrast, passive application of 37% H₃PO₄ or its use with a sponge resulted in significantly lower surface loss. These results reinforce that both the type of acid and the application way influence surface removal — a key concern addressed in the current study.

In this context, the results highlight the need for caution when actively applying stronger acids such as 15% HCl. The enamel loss observed under these conditions exceeded not only the typical thickness of the pseudointact surface layer but also the depth of many early lesions, which reinforces the importance of balancing surface etching with tissue preservation in microinvasive treatments.

Conversely, the acid application way seems to have limited influence on the general surface morphology. Scanning electron microscopy (SEM) analysis did not show any association between enamel treatment and the different micromorphological etching patterns. Type II patterns predominated across all groups, except for 1xA, indicating similar surface characteristics regardless of the application methods and numbers of applications. Therefore, the third null hypothesis was not rejected.

Arnold, et al.^2 1^ (2015) attributed the variations in etching patterns following multiple applications of 15% HCl primarily to intrinsic differences in enamel prism morphology, rather than to the etching protocol itself. This interpretation supports the recent findings, which suggests that the micromorphological similarities across treatment groups were more likely governed by enamel structural characteristics than by the mode or frequency of acid application.

Notably, in the previously discussed study by Yim, et al.^39^(2014), different acid application protocols resulted in distinct surface morphologies on enamel artificial lesions. Passive application of 15% HCl for 120 s produced evident clefts and grooves, yielding a rougher surface. In contrast, 37% H₃PO₄ applied passively or with a sponge for 30 s resulted in less defined features and surfaces lacking the porosity typically required for effective resin infiltration. However, when the same acid was applied mechanically using a brush, the surface appeared relatively smooth but had numerous openings. These findings highlight that the application way — particularly the introduction of friction — may exert a more significant influence on surface morphology than acid alone. Brush application is presumed to generate greater friction than sponge use, leading to more pronounced surface morphology changes.^39^

Clinically, the enamel etching pattern does not seem to be a critical determinant of successful treatment.^40^ However, in cases of over-etching, alterations in etching patterns have been shown to have detrimental effects,^35^ which leads to collapsed structures that are less conducive to resin infiltration.^36^

Given these and other clinical nuances, it is important to acknowledge that this study employed artificial lesions produced in bovine enamel, which differ from natural white spot lesions in terms of mineral content, structure, and lesion depth. While such *in vitro* models are widely used due to their practicality and reproducibility, accurately simulating the complex characteristics of natural carious lesions — particularly those with greater depth — continues to represent a significant and widely acknowledged challenge in laboratory research. Special attention was devoted to validating lesion characteristics using transverse microradiography (TMR), the gold-standard method for assessing mineral loss and lesion depth, to mitigate this limitation and enhance the relevance of the model. Even so, the results should be interpreted as insights into the effects of acid application methods on enamel surface loss rather than definitive clinical recommendations.

Within this context, these findings highlight the importance of adhering to passive, manufacturer-recommended protocols for 15% HCl application in the treatment of white spot lesions. Passive etching, applied one to three times, resulted in no statistically significant differences in surface loss, which supported its relatively conservative and effective use. Conversely, active acid application substantially increased enamel removal, reaching levels comparable to microabrasion, thus posing a risk to enamel integrity and challenging the microinvasive principles of resin infiltration therapy. These results underscore the clinical imperative to balance effective resin penetration with preservation of enamel structure, favoring minimal and controlled etching protocols.

## Conclusion

HCl active application resulted in significantly greater surface loss than passive application, and repeated applications further amplified this effect. In any case, distinct micromorphological etching patterns were not associated with the different treatment protocols. These results suggest that multiple and active applications of HCl could potentially compromise the preservation of residual tooth structure, thereby conflicting with the objectives of minimal intervention dentistry.

Statement of ethics: The study protocol was submitted to the Ethics Committee on Animal Use of the University of São Paulo School of Dentistry, and received an exemption from ethical review under protocol number 004/2023.
